# *Trypanosoma cruzi* XRNA granules colocalise with distinct mRNP granules at the nuclear periphery

**DOI:** 10.1590/0074-02760170531

**Published:** 2018-06-18

**Authors:** Jimena Ferreira da Costa, Mariana Galvão Ferrarini, Sheila Cristina Nardelli, Samuel Goldenberg, Andréa Rodrigues Ávila, Fabíola Barbieri Holetz

**Affiliations:** Fundação Oswaldo Cruz-Fiocruz, Instituto Carlos Chagas, Curitiba, PR, Brasil

**Keywords:** XRNA, mRNP granules, nuclear periphery, metacyclogenesis

## Abstract

**BACKGROUND:**

Eukaryotic ribonucleoprotein (RNP) granules are important for the regulation of RNA fate. RNP granules exist in trypanosomatids; however, their roles in controlling gene expression are still not understood. XRNA is a component of granules in *Trypanosoma brucei* but has not been investigated in *Trypanosoma cruzi.*

**OBJECTIVES:**

This study aimed to investigate the TcXRNA dynamic assembly and its interaction with RNP components under conditions that affect the mRNA availability.

**METHODS:**

We used *in vitro* metacyclogenesis of *T. cruzi* to observe changes in RNP granules during the differentiation process. TcXRNA expression was analysed by Western blot and immunofluorescence. Colocalisation assays were performed to investigate the interaction of TcXRNA with other RNP components.

**FINDINGS:**

TcXRNA is constantly present during metacyclogenesis and is localised in cytoplasmic granules. TcXRNA does not colocalise with TcDHH1 and TcCAF1 granules in the cytoplasm. However, TcXRNA granules colocalise with mRNP granules at the nuclear periphery when mRNA processing is inhibited.

**MAIN CONCLUSIONS:**

TcXRNA plays a role in mRNA metabolism as a component of mRNP granules whose assembly is dependent on mRNA availability. TcXRNA granules colocalise with distinct RNP granules at the nuclear periphery, suggesting that the perinuclear region is a regulatory compartment in *T. cruzi* mRNA metabolism.

Trypanosomatids, such as *Trypanosoma brucei*, *Trypanosoma cruzi* and *Leishmania*, are pathogenic protozoa that account for the deaths of thousands of people every year (DNDi - Available from: http://www.dndial.org/pt/doencas-negligenciadas.html). These parasites have evolved unusual mechanisms of gene expression that are distinct from other eukaryotes (Kramer 2012). A canonical promoter for transcription initiation by RNA polymerase II is not present in their genomes, and protein-coding genes are transcribed into polycistronic RNA precursors, which are further processed into individual mRNAs by *trans-splicing* and polyadenylation (Liang et al. 2003). For this reason, gene expression is regulated mainly at the post-transcriptional level since transcription initiation is not the major rate-limiting factor for mRNA production. Mechanisms that affect RNA stability, RNA localisation or mRNA ribosome occupancy can determine the fate of mRNA (Kramer 2012).

In eukaryotic cells, ribonucleoprotein (RNP) granules are important for the regulation of RNA fate. In somatic cells, P-bodies (PBs) and stress granules (SGs) are the most important and well-studied RNP granules (Buchan 2014). These granules contain mRNPs (messenger ribonucleoprotein) that are not committed to translation and may use similar mechanisms to regulate mRNA metabolism. SGs are induced upon cellular stress, while PBs are constitutively present in the cell. For both types of granules, the assembly dynamics, number and size are all dependent on the translation status (Kedersha and Anderson 2009). In general, PBs are sites where mRNA can be degraded or stored for subsequent return to polysomes, while SGs are involved in the sorting and storage of various transcripts (Kedersha et al. 2005). In fact, there is a dynamic relationship between SGs and PBs, in which selective mRNAs may move from one granule to another (Brengues et al. 2005). Each type of RNP granule has many specific protein markers. However, many proteins, including XRN1, DHH1, eIF4E, and APOBEC3G, have been described as part of both granules (Poblete-Durán et al. 2016).

RNP granules were first described in trypanosomatids in studies in *T. cruzi* and *T. brucei*. These RNP granules contain polyadenylated mRNAs and several RNA-binding proteins, including DHH1, XRNA (yeast and human XRN1 homologue), SCD6, PABP1 and eIF4E (Cassola et al. 2007, Holetz et al. 2007). It was proposed that *T. cruzi* DHH1 granules are similar to PBs due to their constitutive presence in replicative cells. Furthermore, the number of bodies increased during repression of translation (Holetz et al. 2007). Granules similar to PBs were also identified in *T. brucei* and contain DHH1, XRNA and SCD6 (Kramer et al. 2008, Cassola 2011).

Although previous studies have reported many types of RNP granules in trypanosomatids, as reviewed by Cassola (2011), the dynamics of RNP granule interaction and their precise role in controlling gene expression are still not fully understood. Based on previous studies, it has been hypothesised that trypanosome RNP granules have a heterogeneous composition of both protein and RNA during their life cycle, and this composition can be altered by stress conditions (Holetz et al. 2010, Romaniuk et al. 2016). Previous works have shown that XRNA is a component of granules in other trypanosomatids. For example, *T. brucei* XRNA is present in both PBs and nuclear periphery granules (Kramer et al. 2008, Kramer et al. 2012). However, in *T. cruzi*, it was demonstrated that XRNA was localised in a speckled cytoplasmic pattern under favourable growth conditions but colocalised with mRNA granules in parasites subjected to starvation stress (Cassola et al. 2007). Furthermore, RNP granules induced by heat-shock stress were observed at the posterior pole of the *T. brucei* cell. The dynamic assembly of these granules was similar to PBs, but the composition of these granules includes XRNA and excludes DHH1 and SCD6 (Kramer et al. 2008).

It is well known that *T. cruzi* differentiation into infective forms is triggered by nutritional stress, and this process can be mimicked *in vitro*. This approach allows for the study of RNP granules in distinct stages of the parasite life cycle (Contreras et al. 1985, Holetz et al. 2007). For this reason, we used *T. cruzi* metacyclogenesis as a model to obtain further insight into the changes of RNP granules during the differentiation process. We further aimed to investigate granule component interaction during the parasite life cycle. Since XRNA is a component of different types of mRNA granules in trypanosomatids, we examined the assembly dynamics of XRNA granules during *T. cruzi* differentiation and evaluated their association with other components. Here, we show that TcXRNA is a protein constantly present during metacyclogenesis and is localised in cytoplasmic granules. The assembly of TcXRNA granules is dynamic and modified by non-translating mRNA availability. In addition, TcXRNA does not colocalise with other granules in the cytoplasm, which suggests the presence of distinct mRNA granules in *T. cruzi*. However, TcXRNA granules colocalise with distinct granules at the periphery of the nucleus, mainly when mRNA processing is inhibited. This finding indicates that the perinuclear region is involved in the regulation of *T. cruzi* mRNA metabolism.

## MATERIALS AND METHODS

*Parasite culture* - The *in vitro* differentiation of *T. cruzi* Dm28c was performed as previously described (Contreras et al. 1985).

*Production of polyclonal antiserum* - The coding regions of the two proteins (GenBank identification number TcDHH1: Tc00.1047053506959.30, Tc*X*RNA: Tc00.1047053505939.89) were amplified by polymerase chain reaction (PCR) using genomic DNA of *T. cruzi* Dm28c as the template [the oligonucleotide sequences are listed in Supplementary data (Table)]. The amplicons were cloned into the pDEST_17 expression vector (Gateway Technology), according to the manufacturer's protocol (http://www.invitrogen.com). Construct purification and transformation as well as recombinant protein induction and purification were performed according to the Ni-NTA Spin Handbook (Qiagen, Hilden, Germany). Anti-*Tc*XRNA antiserum was produced in mice, while Anti-*Tc*Dhh1 antiserum was produced in rabbits.

*Transfection of parasites with pTcGFP vectors* - Coding sequences of *Tc*XRNA (Tc00.1047053505939.89 and Tc00.1047053507817.80) and *Tc*Caf1 (Tc00.1047053511827.60) genes were amplified by PCR using Dm28c genomic DNA as the template [the oligonucleotide sequences are listed in Supplementary data (Table)]. The amplified genes were inserted into a p*Tc*GFP vector from the pDONR221 vector (Gateway Technology - Invitrogen, Carlsbad, USA), as previously described (Batista et al*.* 2010). *T. cruzi* epimastigotes (5 × 10^8^ cells) were harvested by centrifugation at 7,000 × *g* for 5 min at room temperature, washed three times in phosphate-buffered saline (PBS) and resuspended in 1 mL of electroporation buffer (140 mM NaCl, 25 mM HEPES, 0.74 mM Na_2_HPO_4_, pH 7.5). Cells were then transferred to a 0.2 cm gap cuvette, and 50 μg of DNA was added. The mixture was kept on ice for 10 min and then subjected to two pulses of 450 V and 500 μF using the Gene Pulser II (Bio-Rad, Hercules, USA). After electroporation, cells were inoculated in 10 mL of LIT medium containing 10% FCS and incubated at 28°C. After 24 h of incubation, 250 μg/mL of antibiotic (Geneticin) was added. Then, 72 h after electroporation, cultures were diluted 1:10, and the antibiotic concentration was increased to 500 μg/mL. Stable resistant cells were obtained approximately 20 to 30 days after transfection and a negative control was used to validate this step. Transfected parasites were used for immunolocalisation of selected proteins.

*Western blot analysis* - For Western blot analysis, 250 μg of protein extracts were run in each lane and transferred to nitrocellulose membranes. For the metacyclogenesis experiments, the membranes were incubated with a 1:250 dilution of the antiserum raised against the recombinant *Tc*XRNA protein. For GFP-expressing parasite validations, the membranes were incubated with a 1:4000 dilution of rabbit anti-GFP antiserum (Invitrogen). Antiserum against *Tc*GAPDH (1:100 dilution), kindly provided by Dr Stênio Perdigão Fragoso (ICC/FIOCRUZ/Brazil), was used as a loading control. The membranes were then washed in PBS/Tween 20 and incubated with IRDye^®^ 680LT goat anti-mouse and IRDye^®^ 800CW goat anti-rabbit antibodies (Bioscience), both diluted 1:15,000. A Li-Cor Biosciences Odyssey scanner was used for fluorescence detection.

*Inhibitor treatments* - Parasites were incubated with puromycin (2 mM, 60 min), cycloheximide (100 μg/mL, 15 min) and sinefungin (2 mg/mL, 30 and 60 min).

*Fluorescence microscopy and image analysis* - Parasites were washed and resuspended at a density of 5 x 10^4^ cells/μL in 4% paraformaldehyde in PBS. Cells were added to poly-L-lysine-coated slides, which were then incubated for 20 min at room temperature. The slides were washed twice with PBS. Fixed cells were permeabilised with 0.1% Triton X-100 in PBS for 5 min and blocked in 5% PBS/BSA for 1 h. After two PBS washes, the cells were then incubated with the primary antiserum for 1 h. Cells were again washed with PBS then incubated with secondary anti-IgG fluorescent-conjugated antibody. The nuclei were marked with DAPI (1:2000 dilution). The dilutions of primary antibodies were as follows: mouse anti-TcXRNA at 1:25, rabbit anti-TcDHH1 at 1:50 and rabbit anti-GFP at 1:500 (Invitrogen). The monoclonal antibody Mab25 (1:1000 dilution), which binds to a *T. cruzi* flagellar calcium-binding protein (Schenkman et al. 1991), was kindly provided by Dr Sergio Schenkman (UNIFESP/Brazil). The dilutions of secondary antibodies were as follows: Alexa 546-conjugated goat anti-mouse antibody at 1:400 (Sigma) and Alexa 488-conjugated goat anti-rabbit antibody at 1:600 (Sigma). Colocalisation images were collected using a confocal Leica TCS SP5 microscope. Alternatively, the images were collected using the Leica DMI6000 B microscope, and fluorescence images were deconvolved using LAS AF *software* - Leica (Leica-microsystems).

*Statistical analysis* - Granules of 100 random cells were counted manually for each experimental condition (metacyclogenesis, puromycin and cycloheximide). Calculations of the mean and standard deviation and Student's T-test were all performed using GraphPad Prism 5 software.

## RESULTS

**Fig. 1:**
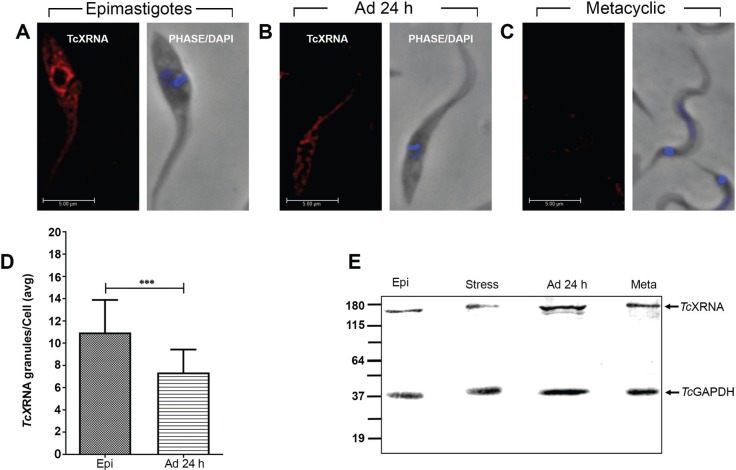
expression and cellular localisation of TcXRNA during *Trypanosoma cruzi* metacyclogenesis. (A) Epimastigotes in logarithmic growth phase. (B) Differentiating epimastigotes (24 h adherent cells). (C) Metacyclic trypomastigotes. Cells were incubated with antiserum against TcXRNA and the immune complexes were detected using Alexa-labelled goat anti-mouse antibodies. Kinetoplasts and nuclei were visualised with DAPI (4',6-diamidino-2-phenylindole). Bars: 5 μm. (D) Bar chart showing the mean number of TcXRNA granules per cell (y-axis) ± standard deviation (n = 100). (***) indicates significant differences (***p < 0.01). (E) Western blot analysis of protein extracts from epimastigotes (Epi), epimastigotes under nutritional stress (Stress), differentiating epimastigotes (cells adherent after 24 h) (Ad 24 h) and metacyclic trypomastigotes (Meta), probed with antiserum against TcXRNA protein (1:250 dilution). The extracts were standardised and all lanes were loaded with 250 μg of protein. TcGAPDH antibody was used as a loading control. The molecular mass marker (in kDa) is the BenchMark Protein Ladder (Invitrogen).

*TcXRNA protein is localised in cytoplasmic granules mainly in replicative epimastigotes* - The expression of TcXRNA was investigated during *T. cruzi* metacyclogenesis by Western blot and immunofluorescence microscopy. A typical cytoplasmic granule fluorescent pattern (Kedersha et al. 2005) was observed for TcXRNA. The granules were distributed throughout the cytoplasm of the replicative epimastigotes with a mean of 10.96 ± 2.9 granules per cell. The granules were concentrated at the nuclear periphery in epimastigotes (Fig. 1A-D). The anti-TcXRNA polyclonal antiserum detected a 162 kDa protein present in all parasite forms analysed (Fig. 1E). However, the number of granules decreased after the induction of metacyclogenesis. Accordingly, differentiating epimastigotes (cells adherent after 24 h) contained 7.37 ± 2.0 granules/cell (Fig. 1B-D). However, the TcXRNA granules are observed in metacyclic trypomastigotes only when we overexposed the time to capture the image [Supplementary data (Fig. 1)]. Thus, the number of granules decreased markedly in fully differentiated metacyclic trypomastigotes, rendering their quantification in this developmental stage difficult (Fig. 1C).

**Fig. 2:**
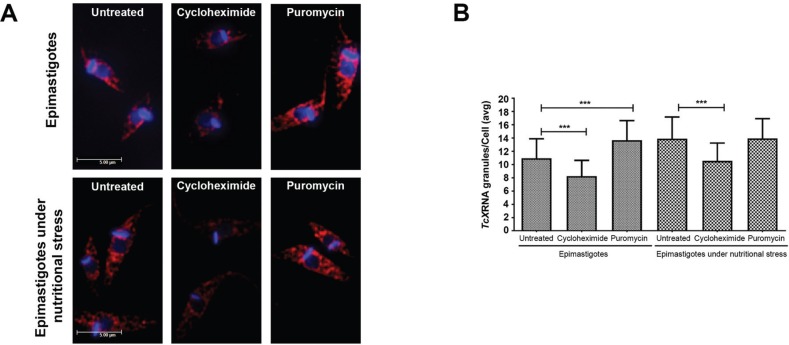
formation of TcXRNA granules after translation process inhibition. (A) Immunolocalisation of TcXRNA in epimastigotes and epimastigotes under nutritional stress. Untreated cells (control), and cells treated with 100 μg/mL cycloheximide or with 2 mM puromycin were incubated with antibody against TcXRNA and detected using Alexa Fluor 546 conjugated secondary antibody. Kinetoplasts and nuclei were visualized using DAPI. The images were collected along a *Z*-series and processed for three-dimensional deconvolution. Bars: 5 μm. (B) Bar chart showing the mean number of TcXRNA granules per cell (y-axis) ± standard deviation (n = 100). (***) indicates significant differences (***p < 0.01).

*The assembly of TcXRNA granules is dynamic and modified by mRNA availability* - The assembly of mRNA granules in *T. cruzi* is dynamic and depends on the availability of mRNA (Cassola et al. 2007, Holetz et al. 2007). Hence, we investigated whether the assembly of TcXRNA granules would be dependent on the translation activity and non-translated mRNA availability (Fig. 2). The results show that the number of TcXRNA granules increased from 10.96 ± 2.9 to 13.91 ± 3.2 granules/cell in epimastigotes under nutritional stress. Furthermore, treatment of epimastigotes with puromycin increased the number of granules from 10.96 ± 2.9 to 13.66 ± 2.9 granules/cell, which may be due to the release of mRNA from polysomes. On the other hand, when mRNAs were stalled in polysomes due to cycloheximide treatment, the number of TcXRNA granules decreased in epimastigotes from 10.96 ± 2.9 to 8.25 ± 2.3. Cycloheximide treatment also affected the assembly of TcXRNA granules in epimastigotes under nutritional stress. In this case, cycloheximide treatment prior to nutritional stress decreased the number of granules from 13.91 ± 3.2 to 10.55 ± 2.6 granules/cell. As expected, treatment with puromycin did not affect the number of granules in parasites under nutritional stress. However, it was observed that TcXRNA granules located at the nuclear periphery were not altered by translation inhibition. These results demonstrate that assembly of cytoplasmic TcXRNA granules is dynamic and is modified by non-translated mRNA availability.

**Fig. 3:**
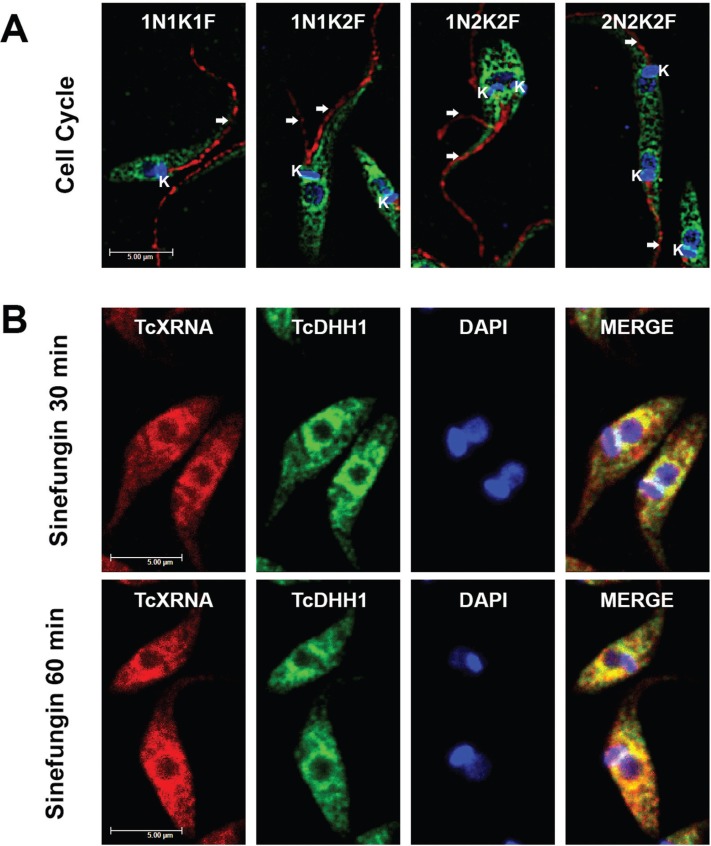
localisation of TcXRNA granules in the nuclear periphery. (A) Tri-dimensional deconvolution images of epimastigote parasites expressing TcXRNAGFP. Parasites were incubated with rabbit anti-GFP (TcXRNA - green) and monoclonal Mab25 (flagellum - red) primary antibodies followed by incubation with Alexa Fluor 488 and Alexa Fluor 546 conjugated secondary antibodies, respectively. DAPI was used to stain the DNA of the nucleus and kinetoplast (blue). Cell cycle phase was determined by the amount of flagella (F), kinetoplasts (K) and nuclei (N) in each parasite. Parasites in the G1 phase have 1N1K1F. In the G2 phase, flagellum duplication occurred (arrows) (1N1K2F). During mitosis, the kinetoplast divided (1N2K2F) and finally, the nucleus divided during cytokinesis (2N2K2F). (B) The effect of sinefungin on TcXRNA and TcDHH1 perinuclear granule formation. Cells were imaged with a confocal microscope 30 and 60 min after sinefungin incubation. Indirect immunofluorescences were performed using antibodies directed to TcXRNA and TcDHH1 followed by incubation with Alexa Fluor 546 and Alexa Fluor 488 conjugated secondary antibodies, respectively. Bars: 5 μm.

*TcXRNA granules are concentrated at the nuclear periphery mainly in the G2 phase of the cell cycle* - Our results demonstrate that TcXRNA granules are present at the nuclear periphery in epimastigotes, and, therefore, we examined whether their presence was correlated with the stages of the cell cycle. The criteria used to define the epimastigote cell cycle stages were the number of flagella (F), the number of kinetoplasts (K) and the number of nuclei (N) (Elias et al. 2009). We observed that TcXRNA was localised at the nuclear periphery at all stages of the cell cycle (Fig. 3A). However, we observed that the distribution of granules at the nuclear periphery was more evident in the G2 phase of the cell cycle (Fig. 3A - 1N1K2F) than the G1/S phase [Fig. 3A - 1N1K1F and Supplementary data (Fig. 2)]. This perinuclear distribution persisted until the end of mitosis (Fig. 3A - 1N2K2F). On the other hand, the TcXRNA granules were dispersed throughout the cytoplasm during late cytokinesis (Fig. 3A - 2N2K2F). This accumulation of granules around the nucleus resembles the nuclear periphery granules described in *T. brucei* by Kramer et al. (2012). In that study, it was concluded that the inhibition of mRNA maturation by sinefungin, a drug that inhibits the trans-splicing mechanism, causes PB proteins to relocalise at the nuclear periphery. Therefore, we analysed the colocalisation of TcXRNA with TcDHH1, a PB marker in *T. cruzi*, after sinefungin treatment [Fig. 3B, Supplementary data (Fig. 3)]. As in *T. brucei*, sinefungin treatment resulted in the accumulation of TcXRNA and TcDHH1 granules at the nuclear periphery after 30 min. However, the granules became dispersed through the cytoplasm if the sinefungin treatment persisted for 60 min. This result indicates that the assembly of TcXRNA granules is dynamic and confirms the association with TcDHH1 granules preferentially at the perinuclear region in *T. cruzi*.

**Fig. 4:**
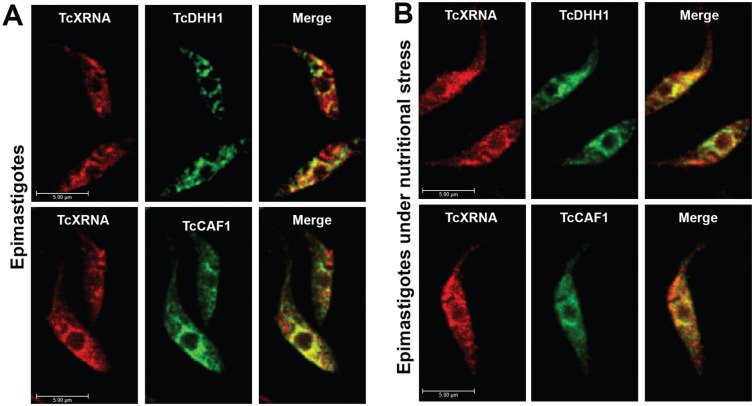
colocalisation analysis of TcXRNA with components of mRNA granules in trypanosomes. (A) Confocal images of epimastigotes. Immunofluorescence studies were performed using mouse and rabbit primary antibodies to TcXRNA and TcDHH1, then epimastigotes were incubated with Alexa Fluor 546 and Alexa Fluor 488 conjugated secondary antibodies, respectively. Epimastigote-transfected parasites expressing TcCAF1GFP were incubated with mouse anti-TcXRNA and rabbit anti-GFP primary antibodies followed by incubation with Alexa Fluor 546 and Alexa Fluor 488 conjugated secondary antibodies, respectively. Merge panels show superimposed images of TcXRNA and TcDHH1 or TcXRNA and TcCAF1. Bars: 5 μm. (B) The experiment was repeated for epimastigotes under nutritional stress.

*TcXRNA granules colocalise with DHH1 and CAF1 at the nuclear periphery* - To provide new insights into TcXRNA granule function, we next determined if the granules colocalised with proteins that are components of mRNA granules in trypanosomes and other eukaryotes. Therefore, we obtained polyclonal antibodies against TcDHH1 protein and cell lines expressing CAF1 protein fused to GFP [Supplementary data (Fig. 4)] to perform colocalisation assays with TcXRNA antibodies. The DEAD-box ATP-dependent helicase Dhh1 stimulates mRNA decay and translation repression and is considered a PB marker in several organisms (Mair et al. 2006, Teixeira and Parker 2007, Presnyak and Coller 2013). The results displayed in Fig. 4 indicate that TcXRNA granules do not colocalise with TcDHH1 granules in the cytoplasm; however, there is an evident colocalisation mainly at the nuclear periphery in both stressed and unstressed epimastigotes (Fig. 4A-B). The quantification of fluorescence intensity confirmed the colocalisation of proteins at the nuclear periphery [Supplementary data (Fig. 3)].

The Ccr4-Not complex includes deadenylating enzymes and is an important regulator of gene expression. In the cytoplasm, it is present in polysomes and in RNA granules where translationally stalled mRNAs are redirected (Collart 2016). CAF1, a catalytic subunit of the Ccr4-Not complex, is present in *T. brucei* (Schwede et al. 2009). We identified a possible homologue of TbCAF1 in *T. cruzi* (TcCAF1) [Supplementary data (Fig. 5)] and demonstrated that this protein was localised in cytoplasmic granules in epimastigotes [Supplementary data (Fig. 4)]. In addition, similarly to TcDHH1, TcCAF1 granules colocalised with TcXRNA granules only at the nuclear periphery in epimastigotes and epimastigotes under nutritional stress (Fig. 4A-B). Taken together, our results demonstrate the colocalisation of TcXRNA with distinct mRNA granules in *T. cruzi*, which occurred mainly at the nuclear periphery. In addition, sinefungin treatment increased the colocalisation of TcDHH1 and TcXRNA granules.

## DISCUSSION

Cytoplasmic mRNP granules are important players in the regulation of gene expression (Kramer 2012), and cytoplasmic XRN1 is the main mRNA decay enzyme present in PBs and SGs in eukaryotic cells (Kedersha et al. 2005). The homologue of XRN1 (TcXRNA) has been identified in cytoplasmic granules of *T. cruzi*, but no further investigation of TcXRNA has been performed during *T. cruzi* differentiation. Here, we demonstrated that TcXRNA is localised in cytoplasmic granules that vary in number during *T. cruzi* differentiation. The number of TcXRNA granules decreases in both differentiating parasites and metacyclic trypomastigotes, despite the constitutive expression of the protein in all *T. cruzi* developmental stages. Although the transcriptional activity is highly reduced in metacyclic (Ferreira et al. 2008), the protein is expressed in these forms. Thus, the number of TcXRNA granules is probably affected by changes in their assembly and/or distribution but is not affected by changes in TcXRNA expression.

We previously demonstrated similar results for TcDHH1 granules (Holetz et al. 2007, Holetz et al. 2010). TcDHH1 expression is not regulated through the parasite life cycle, and this protein is located in cytoplasmic foci that vary in number according to nutritional stress conditions and cycloheximide/puromycin treatment. In general, the assembly of cytoplasmic mRNA granules is triggered by processes that lead to polysome dissociation with the corresponding association of the released mRNAs in granules (Brengues et al. 2005). Therefore, we investigated if TcXRNA granules assembly could be modified by conditions that alter the availability of mRNA, as polysome dissociation. In our analysis, the number of TcXRNA granules increases under nutritional stress, which interferes with the abundancy of polysomes. It is known that nutritional stress results in triggering *T. cruzi* differentiation, and it has been observed that compared to those from growing cells, starved parasites present shorter and less abundant polysomes (Holetz et al. 2007, Alves et al. 2010), and we believed that the TcXRNA granule assembly increases due to the release of non-translated mRNA from polysomes. Indeed, the treatment of cells with puromycin, which inhibits the translation process by releasing mRNAs from polysomes, also increased the number of TcXRNA granules, while the treatment with cycloheximide, which inhibits translation by trapping mRNAs to polysomes, decreased the number of TcXRNA granules in epimastigotes. Thus, the data strongly suggest that TcXRNA assembly is increased by the availability of non-translated mRNA. Furthermore, cycloheximide treatment prior to nutritional stress prevented polysome dissociation, and the number of TcXRNA granules decreased. Not surprisingly, puromycin treatment prior to nutritional stress did not change the number of TcXRNA granules. These results demonstrate that the release of mRNAs from polysomes is necessary to increase the assembly of cytoplasmic TcXRNA granules. Since PBs are present in unstressed cells and their assembly is dependent on and proportional to the pool of nontranslated mRNAs (Teixeira and Parker 2007), we confirm that TcXRNA protein is an important constituent of granules similar to PBs in *T. cruzi*.

We also observed that TcXRNA granules accumulate at the nuclear periphery in epimastigotes, remarkably during G2 phase of the cell cycle. Perinuclear germ granules were described in *C*. *elegans* associated with the nuclear pore complexes, possibly related to mRNA regulatory mechanisms (Sheth et al. 2010). Granules at the nuclear periphery (NPGs) have also been observed in *T. brucei*, and they are dependent on the integrity of the nuclear pore complexes and accumulate at the perinuclear region as a result of mRNA processing inhibition (Kramer 2012). It is believed that NPGs act as sorting compartments to determine the fate of mRNAs in *T. brucei*. Accordingly, our results show that inhibition of mRNA processing by sinefungin induced the accumulation of TcXRNA granules at the nuclear periphery in epimastigotes. We speculate that perinuclear granule assembly may be induced by the lack of processed mRNA. It is not clear if there is a functional (Non-sense-mediated decay - NMD) system in trypanosomes (Delhi et al. 2011), and recent work suggests a possible divergence within mRNA export mechanisms with the absence of the major mRNA maturation platform at the cytoplasmic face of the nuclear pore complex (Obado et al. 2016). Thus, we can speculate that the perinuclear distribution of TcXRNA granules might be related to a potential role in mRNA sorting in *T. cruzi*.

Interestingly, the perinuclear distribution of TcXRNA granules is not affected by translation inhibition. Therefore, the formation of TcXRNA perinuclear granules responds to changes in gene expression in a different manner than TcXRNA dispersed cytoplasmic granules. Since, the assembly of perinuclear and dispersed cytoplasmic granules of TcXRNA seems to be modified by different signals we can speculate that this would occur due to the association of TcXRNA with different sets of proteins. It is well accepted that the protein composition of granules defines their function (Buchan 2014). In general, SGs are characterised by the presence of translation initiation factors and small ribosomal subunit proteins, whereas PBs are defined by the presence of proteins involved in mRNA decay and translation repression (Kedersha and Anderson 2009). To get further insight into the dynamics of RNA granules in *T. cruzi*, we performed colocalisation assays between TcXRNA and TcDHH1 and TcCAF1, since they are involved in different mechanisms of gene expression control. TcDHH1 is localised to discrete cytoplasmic foci that regulate the expression of stage-specific genes (Holetz et al. 2007, Holetz et al. 2010). CAF1 is the major deadenylase in *T. brucei* and is involved in the turnover of several constitutively expressed or long-lived mRNAs (Schwede et al. 2009, Fadda et al. 2014). As yet, CAF1-NOT complex activity has not been studied in *T cruzi*. Our results show that TcXRNA granules do not colocalise with TcDHH1 or TcCAF1 in the cytoplasm of parasites in the tested conditions. Although nutritional stress increased the number of cytoplasmic granules, we did not observe an evident colocalisation between TcXRNA and TcDHH1 proteins in the cytoplasm. Intriguingly, in the case of *T. brucei*, previous work indicated that XRNA and DHH1 localised in the same cytoplasmic foci when the parasites were treated with puromycin (Kramer et al. 2008). In addition, it was observed that heat shock (41°C) induced the appearance of a novel focus at the posterior pole of the cell, which contained XRNA but not DHH1 and other PB proteins such as SCD6. In contrast, we did not observe the assembly of a posterior pole granule containing TcXRNA in *T. cruzi* under any of the conditions analysed in this study. Our data suggests that TcXRNA and TcDHH1 are components of distinct granules. Hence, the assembly of cytoplasmic TcXRNA granules in *T. cruzi* and in *T. brucei* might be differently modulated.

On the other hand, both TcDHH1 and TcCAF1 colocalised with TcXRNA at the nuclear periphery. In the nucleus of various eukaryotic species, Ccr4 and Not4 (from the Ccr4/Caf1/Not complex) are important for mRNA nuclear quality control and export, as reviewed by Collart (2016). However, no Ccr4 or Not4 homologues were identified in trypanosomes, and the CAF1-NOT complex is simpler as compared to other organisms (Erben et al. 2014). In addition, TcCAF1 is not a nuclear protein, which indicates that CAF1-NOT-dependent nuclear quality control might not be functional in trypanosomatids. Thus, we believe that an important mRNA quality control checkpoint occurs at the nuclear periphery of *T. cruzi* cells and involves the association of distinct proteins, such as TcXRNA, TcDHH1 and TcCAF1.

In conclusion, TcXRNA seems to play a role in *T. cruzi* mRNA metabolism, as a component of mRNP granules whose assembly is dependent on mRNA availability. In addition, TcXRNA granules do not colocalise with TcDHH1 and TcCAF1 granules dispersed in the cytoplasm, indicating that they are components of distinct cytoplasmic granules. However, these proteins colocalise at the nuclear periphery, leading to the suggestion of a regulatory compartment involving the activity of mRNP granules at the nuclear periphery in *T. cruzi*.

## References

[B1] Alves LR, Ávila AR, Correa A, Holetz FB, Mansur FCB, Manque PA (2010). Proteomic analysis reveals the dynamic association of proteins with translated mRNAs in Trypanosoma cruzi. Gene.

[B2] Batista M, Marchini FK, Celedon PF, Fragoso SP, Probst CM, Preti H (2010). A high-throughput cloning system for reverse genetics in Trypanosoma cruzi. BMC Microbiol.

[B3] Brengues M, Teixeira D, Parker R (2005). Movement of eukaryotic mRNAs between polysomes and cytoplasmic processing bodies. Science.

[B4] Buchan JR (2014). mRNP granules. Assembly, function, and connections with disease. RNA Biol.

[B5] Cassola A, de Gaudenzi JG, Frasch AC (2007). Recruitment of mRNAs to cytoplasmic ribonucleoprotein granules in trypanosomes. Mol Microbiol.

[B6] Cassola A (2011). RNA granules living a post-transcriptional life: the trypanosomes' case. Curr Chem Biol.

[B7] Collart MA (2016). The Ccr4-Not complex is a key regulator of eukaryotic gene expression. Wiley Interdiscip Rev RNA.

[B8] Contreras VT, Salles JM, Thomas N, Morel CM, Goldenberg S (1985). In vitro differentiation of Trypanosoma cruzi under chemically defined conditions. Mol Biochem Parasitol.

[B9] Delhi P, Queiroz R, Inchaustegui D, Carrington M, Clayton C (2011). Is there a classical nonsense-mediated decay pathway in trypanosomes?. PLoS One.

[B10] Elias MC, Nardelli SC, Shenckman S (2009). Chromatin and nuclear organization in Trypanosoma cruzi. Future Microbiol.

[B11] Erben E, Chakraborty C, Clayton C (2014). The CAF1-NOT complex of trypanosomes. Front Genet.

[B12] Fadda A, Ryten M, Droll D, Rojas F, Färber V, Haanstra JR (2014). Transcriptome-wide analysis of trypanosome mRNA decay reveals complex degradation kinetics and suggests a role for co-transcriptional degradation in determining mRNA levels. Mol Microbiol.

[B13] Ferreira L, Dossin F, Ramos T, Müller E, Schenkman S (2008). Active transcription and ultrastructural changes during Trypanosoma cruzi metacyclogenesis. An Acad Bras Cienc.

[B14] Holetz FB, Alves LR, Probst CM, Dallagiovanna B, Marchini FK, Manque P (2010). Protein and mRNA content of TcDHH1-containing mRNPs in Trypanosoma cruzi. FEBS J.

[B15] Holetz FB, Correa A, Avila AR, Nakamura CV, Krieger MA, Goldenberg S (2007). Evidence of P-body-like structures in Trypanosoma cruzi. Biochem Biophys Res Commun.

[B16] Kedersha N, Anderson P (2009). Regulation of translation by stress granules and processing bodies. Prog Mol Biol Transl Sci.

[B17] Kedersha N, Stoecklin G, Ayodele M, Yacono P, Lykke-Andersen J, Fritzler MJ (2005). Stress granules and processing bodies are dynamically linked sites of mRNP remodeling. J Cell Biol.

[B18] Kramer S, Marnef A, Standart N, Carrington M (2012). Inhibition of mRNA maturation in trypanosomes causes the formation of novel foci at the nuclear periphery containing cytoplasmic regulators of mRNA fate. J Cell Sci.

[B19] Kramer S, Queiroz R, Ellis L, Webb H, Hoheisel JD, Clayton C (2008). Heat shock causes a decrease in polysomes and the appearance of stress granules in trypanosomes independently of eIF2(alpha) phosphorylation at Thr169. J Cell Sci.

[B20] Kramer S (2012). Developmental regulation of gene expression in the absence of transcriptional control: the case of kinetoplastids. Mol Biochem Parasitol.

[B21] Liang X, Haritan A, Uliel S, Michaeli S (2003). Trans and cis splicing in trypanosomatids : mechanism, factors, and regulation. Eukaryotic Cell.

[B22] Mair GR, Braks JAM, Garver LS, Wiegant JCAG, Hall N, Dirks RW (2006). Regulation of sexual development of Plasmodium by translational repression. Science.

[B23] Obado SO, Brillantes M, Uryu K, Zhang W, Ketaren NE, Chait BT (2016). Interactome mapping reveals the evolutionary history of the nuclear pore complex. PLoS Biol.

[B24] Poblete-Durán N, Prades-Pérez Y, Vera-Otarola J, Soto-Rifo R, Valiente-Echeverría F (2016). Who regulates whom? An overview of RNA granules and viral infections. Viruses.

[B25] Presnyak V, Coller J (2013). The DHH1/RCKp54 family of helicases: an ancient family of proteins that promote translational silencing. Biochim Biophys Acta.

[B26] Romaniuk MA, Cervini G, Cassola A (2016). Regulation of RNA binding proteins in trypanosomatid protozoan parasites. World J Biol Chem.

[B27] Schenkman S, Diaz C, Nussenzweig V (1991). Attachment of Trypanosoma cruzi trypomastigotes to receptors at restricted cell surface domains. Exp Parasitol.

[B28] Schwede A, Manful T, Jha B, Helbig C, Bercovich N, Stewart M (2009). The role of deadenylation in the degradation of unstable mRNAs in trypanosomes. Nucleic Acids Res.

[B29] Sheth U, Pitt J, Dennis S, Priess JR (2010). Perinuclear P granules are the principal sites of mRNA export in adult C. elegans germ cells. Development.

[B30] Teixeira D, Parker R (2007). Analysis of P-body assembly in Saccharomyces cerevisiae. Mol Biol Cell.

