# Supplementary material for: Trypanosoma cruzi XRNA granules colocalise with distinct mRNP granules at the nuclear periphery
Source: Mem Inst Oswaldo Cruz. 2018 Jun 18;113(6):e170531. doi: 10.1590/0074-02760170531 (PMC6001583; doi:10.1590/0074-02760170531)
Supplement: Supplementary file 1 [file 0074-0276-mioc-113-06-e170531-Suppl01.pdf]

TABLE

Oligonucleotide sequences

Gene	Production	Oligo	Sequences
*Tc*XRNA	Antiserum	F	5' GGAGTTCCAAAGTTTTTTCGGTG 3'
		R	5' ACAAATTCATCGGCTTTACGGT 3'
	GFP parasites	F	5' ATGGGAGTTCCAAAGTTTTTTCGGTG 3'
		R	5' CCTATGCGTTGTTGTCTTGC 3'
*Tc*DHH1	Antiserum	F	5' ATGTCTTTGGAAGACGACTG 3'
		R	5' TGCGGCGTAGAGTTCAG 3'
*Tc*Caf1	GFP parasites	F	5' ATGTCAAACACATGGGATTTTATGGAC 3'
		R	5 'GCCCTTCACCGAGTTGTTGAGC 3'

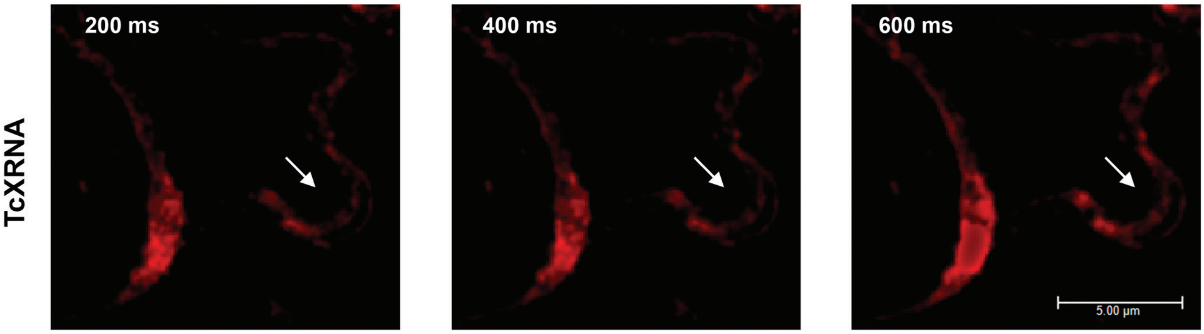

Fig. 1: localisation of TcXRNA granules in epimastigote and metacyclic trypomastigote in different exposure times. Parasites were incubated with antiserum against TcXRNA and the immune complexes were detected using Alexa Fluor 546 goat anti-mouse antibodies. The immunofluorescence images were captured in different exposure times. TcXRNA is observed in metacyclic trypomastigote (arrow) after an overexposure time in comparison with epimastigote images. Bar = 5 µm.

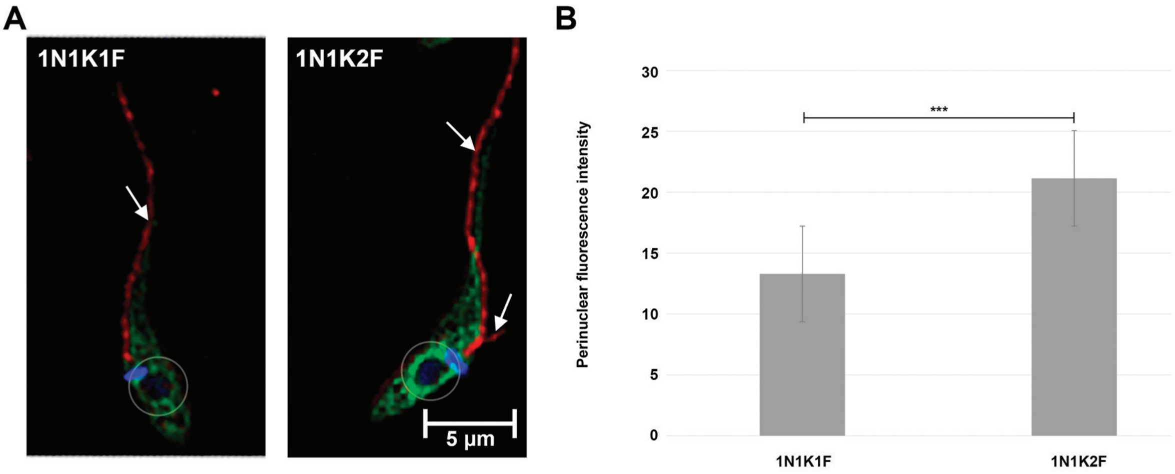

Fig. 2: quantification of perinuclear TcXRNAGFP fluorescence intensity during epimastigote cell cycle. (A) Tri-dimensional deconvolution images of epimastigote parasites expressing TcXRNAGFP. Cells in G1/S (1N1K1F) and in G2 (1N1K2F) are indicated. Parasites were incubated with rabbit anti-GFP and monoclonal Mab25 primary antibodies followed by incubation with Alexa Fluor 488 and Alexa Fluor 546 conjugated secondary antibodies, respectively. DAPI was used to stain the DNA of the nucleus and kinetoplast. Arrows indicate the flagella. Bar = 5 µm. (B) Perinuclear regions were delimited and 25 images of parasites in both G1/S and G2 cell cycle phases were used to quantify the fluorescence intensity by ImageJ software. The ***p<0.001 indicates that the difference between the TcXRNA perinuclear intensity in G1/S and G2 is statistically significant.

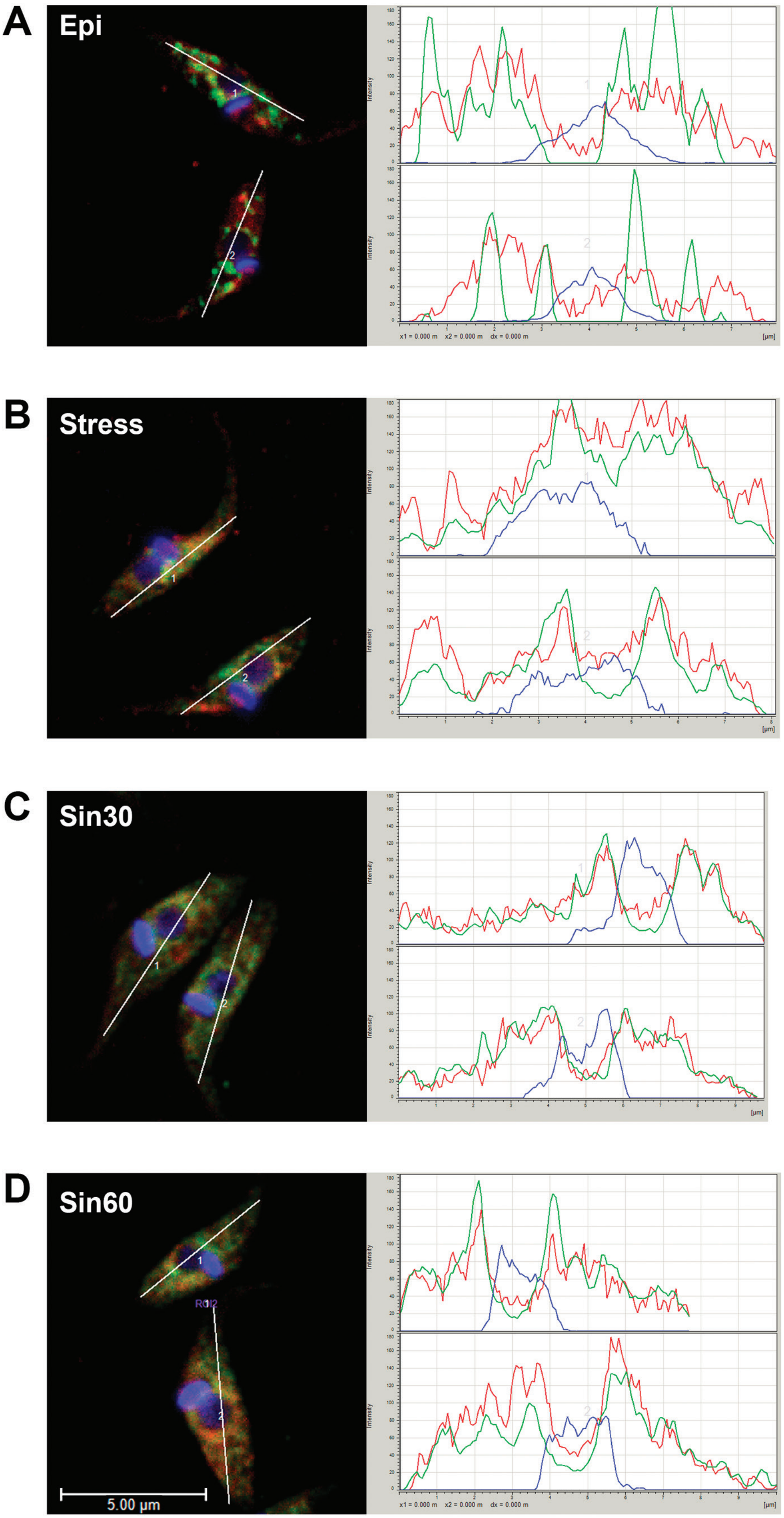

Fig. 3: quantification of fluorescence intensity in images of TcXRNA and TcDHH1 colocalisation. Cells were incubated with primary antibodies against TcXRNA and TcDHH1 followed by incubation with Alexa Fluor 546 and Alexa Fluor 488 conjugated secondary antibodies, respectively. DNA was stained with DAPI. Images were processed by deconvolution software Leica AF6000. The fluorescence intensity was measured in one stack of confocal images of (A) epimastigotes in logarithmic growth phase, (B) epimastigotes under nutritional stress, (C) epimastigotes treated with sinefungin for 30 min, and (D) 60 min. Graphics show the quantification of fluorescence intensity of DAPI (blue), TcXRNA (red) and TcDHH1 (green) fluorescent-labelling along the delimited lines. Fluorescence intensity was plotted on the y-axis. The comparison with DAPI distribution confirms a higher intensity of either green or red fluorescence at nuclear periphery. In addition, it is also possible to observe a similar pattern of fluorescence distribution at the periphery of the nucleus, although the total intensity between green and red fluorescence is not very similar. Bar: 5 µm.

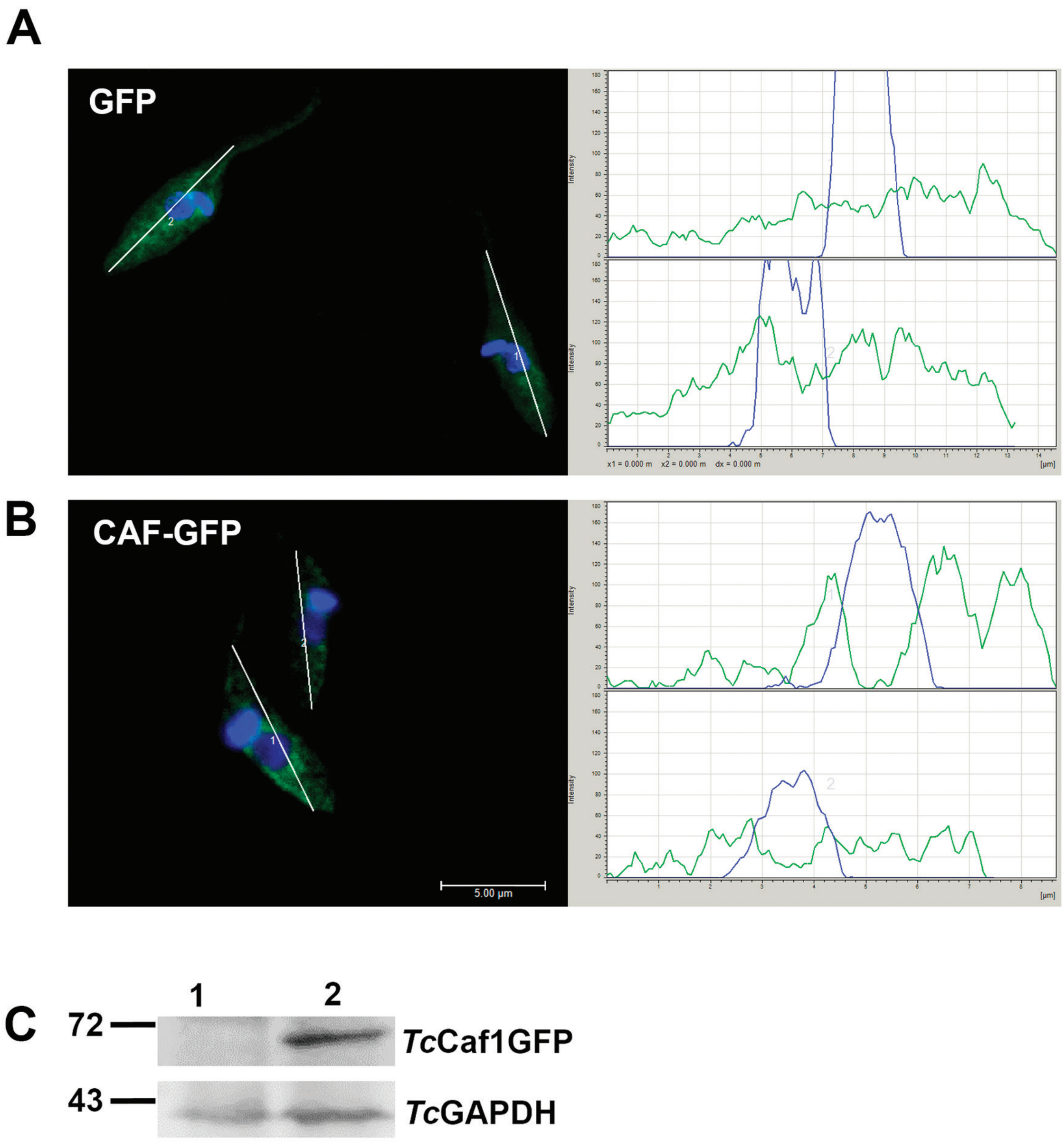

Fig. 4: analysis of parasites expressing TcCAF1 tagged with GFP (TcCAF1GFP). Confocal images of epimastigote-transfected parasites expressing GFP (A) and TcCAF1GFP (B). Parasites were incubated with rabbit anti-GFP primary antibody followed by incubation with Alexa Fluor 488 conjugated secondary antibody. DNA was stained with DAPI. Images were processed by deconvolution software Leica AF6000. Graphs show the quantification of fluorescence intensity of DAPI- (blue) and GFP-labelling (green) along delimited lines. Fluorescence intensity was plotted on the y-axis. Higher fluorescence intensity at the perinuclear region is observed only in the parasite expressing TcCAF1GFP. Bar: 5 µm. (C) Western blots of protein extracts from transfected epimastigotes using anti-GFP monoclonal antibody. The antibody detected the GFP-tagged TcCAF1 protein (64 kDa), corresponding to TcCAF1 (37 kDa) fused to GFP (26 KDa). TcGAPDH antibody was used as a loading control.

```
Score            Expect    Identities       Positives        Gaps
568 bits(1463)   0.0       277/352(79%)     309/352(87%)     17/352(4%)

TRYCR  1    MMQYGNALAQYGTYQQ-----------RYP-VAVSQGGIAAIPSLSKSPMIRDVWEENLE  48
            MMQYG     YG Y Q           R+P  A+  G ++ IPSLSKSPMIRDVWE+NLE
TRYB2  1    MMQYGGG-THYGAYAQQQPQPQPQPHQRFPSAALKAGNVSLIPSLSKSPMIRDVWEDNLE  59

TRYCR  49   EEFNIIRSLIKDYPYVSMDTEFPGVVAKPVGNFKATHEFYYQTLRCNVNLLKMIQLGITL  108
            +EF IIRSLIKDYP+V+MDTEFPGVVAKPVGNFK+THEFYYQTLRCNVNLLKMIQLGITL
TRYB2  60   QEFGIIRSLIKDYPFVAMDTEFPGVVAKPVGNFKSTHEFYYQTLRCNVNLLKMIQLGITL  119

TRYCR  109  LNEKGEVPESCCTWQFNFRFCLTEDVYAQDSIQLLRHGGIDFDYFAQYGVEVTHFAELLI  168
            LNEKGEVPE+CCTWQFNFRFCLTEDVYAQDSIQLL HGGI+FDYF++YGVEVTHFAELLI
TRYB2  120  LNEKGEVPENCCTWQFNFRFCLTEDVYAQDSIQLLCHGGINFDYFSEYGVEVTHFAELLI  179

TRYCR  169  SSGLVLNSDIRWLAFHAGYDFGYLMKVVCGKDLPEKEDDFLQIFHSLFPCVYDIKYLLRA  228
            SSGLVLN DIRWLAFHAGYDFGYL+KVV GKDLPEKE+DFLQ FH+LFPCVYDIKYLLR+
TRYB2  180  SSGLVLNPDIRWLAFHAGYDFGYLIKVVGGKDLPEKEEDFLQTFHALFPCVYDIKYLLRS  239

TRYCR  229  TDLSHSLGLDHLSESLRVRRFGMAHQAGSDSLLTGHCYFKLLRDCFSSNPPVASNGVLYG  288
            T+L+HSLGLDHL++SLRVRRFGMAHQAGSDSLLTGHCYFKLLRDCF+SN PVA+NGVLYG
TRYB2  240  TELTHSLGLDHLADSLRVRRFGMAHQAGSDSLLTGHCYFKLLRDCFNSNIPVANNGVLYG  299

TRYCR  289  LCEDASSAATPSSTTIP---GGSAHAFASFTASKNTTFPSTP-LNHSAKGHN  336
            LCED+SSA TP+STTIP   GG  HAF++FT SKN  FP++P +  + KGH+
TRYB2  300  LCEDSSSAGTPNSTTIPGSGGGGHAFSNFTGSKNAAFPASPIMTQAVKGHS  351
```

Fig. 5: conservation analysis of CCR4-associated factor 1 (CAF1) from *Trypanosoma cruzi* and *Trypanosoma brucei*. BLAST protein alignment between CAF1 from *T. brucei brucei* TREU927 (TRYB2, TrytripID Tb927.6.600) and putative CAF1 from *T. cruzi* Dm28c (TRYCR, TrytripID TCDM_08618) shows that the three proposed regions essential for deadenylase activity (in red) are conserved in *T. cruzi* Dm28c.
